# IGF1R depletion facilitates *MET*-amplification as mechanism of acquired resistance to erlotinib in HCC827 NSCLC cells

**DOI:** 10.18632/oncotarget.16350

**Published:** 2017-03-18

**Authors:** Dianna Hussmann, Anne Tranberg Madsen, Kristine Raaby Jakobsen, Yonglun Luo, Boe Sandahl Sorensen, Anders Lade Nielsen

**Affiliations:** ^1^ Department of Biomedicine, Aarhus University, Aarhus, Denmark; ^2^ Department of Clinical Biochemistry, Aarhus University Hospital, Aarhus, Denmark

**Keywords:** NSCLC, IGF1R, EGFR-TKI, EMT, MET

## Abstract

EGFR-mutated non-small cell lung cancer patients experience relapse within 1-2 years of treatment with EGFR-inhibitors, such as erlotinib. Multiple resistance mechanisms have been identified including secondary *EGFR*-mutations, *MET*-amplification, and epithelial-mesenchymal transition (EMT). Previous studies have indicated a role of Insulin-like growth factor 1 receptor (IGF1R) in acquired resistance to EGFR-directed drugs as well as in EMT. In the present study, we have investigated the involvement of IGF1R in acquired high-dose erlotinib resistance in the *EGFR*-mutated lung adenocarcinoma cell line HCC827. We observed that IGF1R was upregulated in the immediate response to erlotinib and hyperactivated in erlotinib resistant HCC827 cells. Resistant cells additionally acquired features of EMT, whereas *MET*-amplification and secondary *EGFR*-mutations were absent. Using CRISPR/Cas9, we generated a HCC827(IGFR1−/−) cell line and subsequently investigated resistance development in response to high-dose erlotinib. Interestingly, HCC827(IGFR1−/−) cells were now observed to specifically amplify the *MET* gene. Additionally, we observed a reduced level of mesenchymal markers in HCC827(IGFR1−/−) indicating an intrinsic enhanced epithelial signature compared to HCC827 cells. In conclusion, our data show that IGF1R have an important role in defining selected resistance mechanisms in response to high doses of erlotinib.

## INTRODUCTION

Acquired resistance to targeted therapies remains a major challenge in lung cancer treatment. Erlotinib, gefitinib, and afatinib are tyrosine-kinase inhibitors (TKIs) targeting the epidermal growth factor receptor (EGFR). Non-small cell lung cancer (NSCLC) patients with tumors harboring *EGFR*-activating mutations highly benefit from EGFR-directed treatment [1, 2]. But, despite initial response, the vast majority of these patients develop drug resistance within 1-2 years of treatment [3]. Several resistance mechanisms have been identified including the T790M secondary EGFR-mutation [4, 5], phenotypic shifts such as epithelial-mesenchymal transition (EMT) [6–9] or small-cell lung cancer transformation [10], and bypass-signaling through other receptor tyrosine kinases (e.g. AXL, HER2, FGFR1, IGF1R, and MET) [11–14]. One of the most common bypass-signaling mechanisms is *MET* gene amplification [15]. *MET*-amplification was first discovered to convey resistance *in vitro* by Engelman *et al*. with subsequent verification in clinical samples [16]. Aberrant activation of the insulin-like growth factor 1 receptor (IGF1R) has been associated with resistance development to EGFR-targeted treatment in both clinical and pre-clinical studies [17–23]. Upon binding of the ligands IGF1 and IGF2, IGF1R primarily signals through the PI3K/AKT and the Ras/MAPK pathways and stimulates cell proliferation and interrupts programmed cell death [24]. The clinical significance of IGF1R expression and activation has been contradicting [25]. Park *et al*. recently performed an analysis of IGF1R protein expression in NSCLC adenocarcinomas [26]. They found that IGF1R expression status had no significant prognostic value in non-mutated *EGFR* tumors. In contrast, high IGF1R expression was significantly associated with inferior progression-free survival (PFS) in tumors harboring *EGFR* activating mutations. This was supported by the findings of Yeo *et al*. reporting IGF1R protein expression as a negative predictor of response to EGFR-directed treatment in *EGFR*-mutated NSCLC patients [27]. *In vitro* prediction of resistance mechanisms is a powerful tool in dissecting the molecular basis of disease progression. In acquired resistance to EGFR-directed treatment, high IGF1R activity has been linked to intrinsic resistance to gefitinib in NSCLC cell lines, and IGF1R shows noteworthy importance in the resistance development in cells under hypoxic conditions [17–19]. In the *EGFR*-mutated PC9 cell line, IGF1R profoundly cross-talked with EMT to mediate drug resistance [28]. A study by Morgillo *et al*. found EMT features and increased IGF1R activation in TKI-resistant cell lines, but IGF1R inhibition had no significant effect on the viability of resistant cells [20]. This suggests a possible importance of IGF1R in initiating the EMT process but a redundancy in maintaining resistance. However, IGF1R was found to have a significant role in maintaining viability in the *EGFR*-mutated cell lines H1975 and PC9, when they acquired resistance to EGFR-TKIs through the T790M mutation [22, 23]. In the present study, we investigate the role of IGF1R in acquired resistance to erlotinib in the *EGFR* exon19del mutated lung adenocarcinoma cell line HCC827. Using CRISPR/Cas9 mediated gene editing we induce a deletion within the *IGF1R* gene and analyze the mechanisms of resistance development in response to high-dose erlotinib treatment.

## RESULTS

### Acquired erlotinib resistance in HCC827 is associated with increased IGF1R expression and receptor hyperactivation

**Figure 1:**
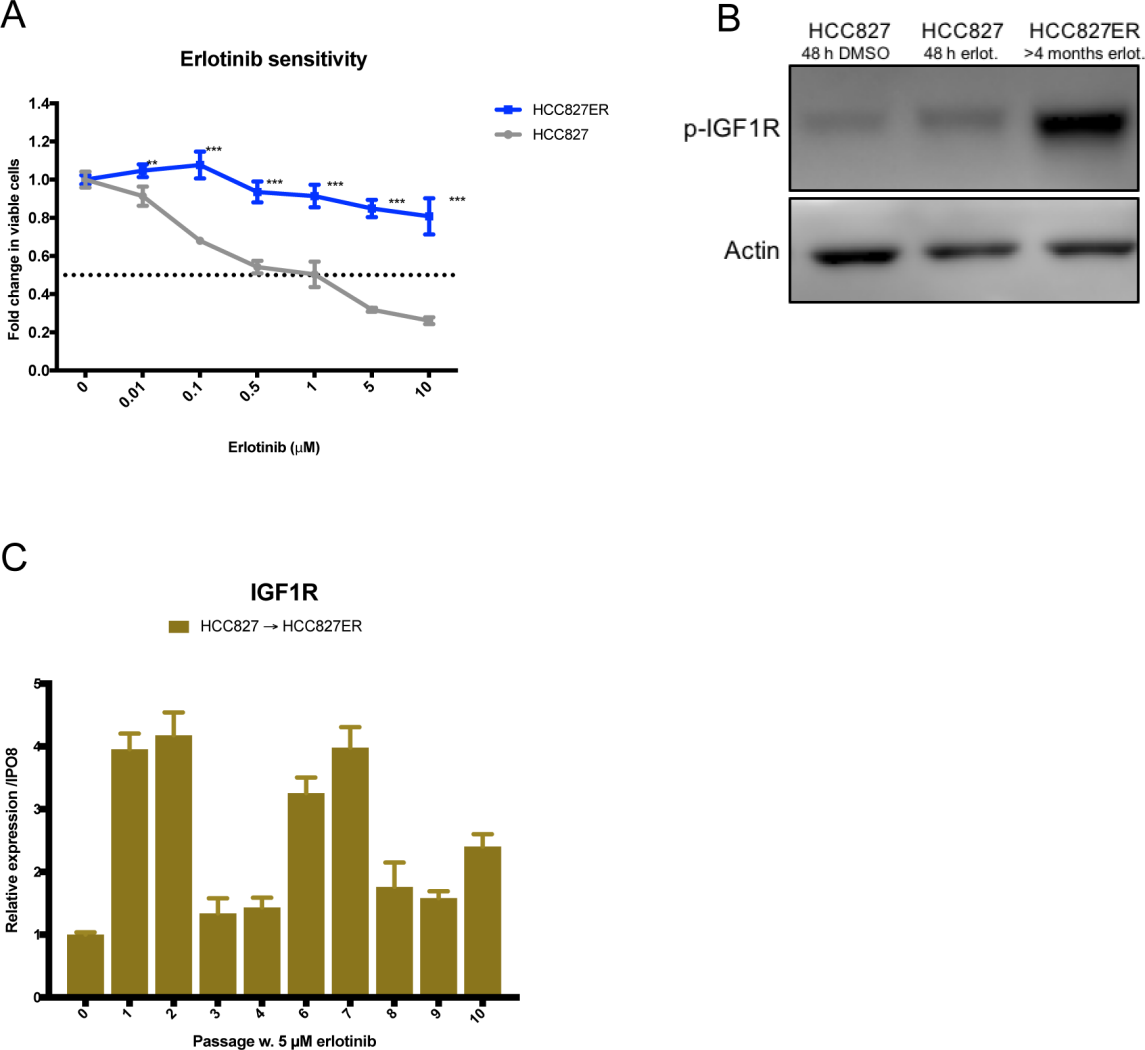
HCC827 acquires resistance to erlotinib by overexpressing and hyperactivating IGF1R **(A)** Viability in response to treatment with increasing concentrations of erlotinib determined by MTS assay in HCC827 and HCC827ER. Parental and erlotinib-resistant cells were treated with indicated concentrations of erlotinib for 72 hours before incubation with MTS solution. All erlotinib dilutions (0-10 μM) were corrected to contain equal amounts of DMSO. The dotted line indicates 0.5-fold change in number of viable cells. The number of viable cells was determined relatively to erlotinib-untreated controls, and fold change in viable cells is plotted as mean ±SD. Significance is calculated for each concentration between parental and resistant cell lines. (*p<0.05, **p<0.01, ***p<0.001). **(B)** Western blot analysis of phosphorylated IGF1R (p-IGF1R) protein expression in HCC827 and HCC827ER. Parental cells were treated with 48 h of DMSO or 5 μM erlotinib prior to protein harvest. β-actin was used as loading control. **(C)** *IGF1R* gene expression changes assessed by qPCR in passage 0 to 10 during resistance development. Gene expression was normalized to *IPO8* and expression levels for each passage were presented relatively to passage 0. Expression levels are based on one biological sample and illustrated as mean ± SD.

Acquired resistance to erlotinib was induced in the highly erlotinib-sensitive HCC827 cell line by continuous high-dose erlotinib treatment. HCC827 erlotinib-resistant cells (HCC827ER) were established after approximately 4 months with 5 μM erlotinib exposure, and the cells no longer responded to erlotinib concentrations up to 10 μM (Figure 1A). In order to determine resistance mechanisms in HCC827ER, we analyzed mutational and signaling changes between parental HCC827 and established HCC827ER cells. Neither the T790M nor other secondary mutations in the *EGFR* gene were detected in HCC827ER, and the cells were found to retain their original exon19 deletion (Supplementary Figure 1A). Receptor phosphorylation screening in a panel of 49 RTKs revealed hyperactivation of IGF1R in HCC827ER (Supplementary Figure 1B), which was further confirmed by western blot analyses (Figure 1B). The RTK screening additionally showed that EGFR signaling was reduced in the resistant cells. Gene expression changes were monitored for each passage throughout resistance development. *IGF1R* gene expression levels were upregulated 2.5-fold in HCC827ER (passage 10) compared to HCC827 (passage 0). In addition, an early 4-fold increase in expression was eminent after 3 weeks of erlotinib exposure (passage 1) (Figure 1C).

### Generation of a HCC827(IGF1R−/−) cell line

**Figure 2:**
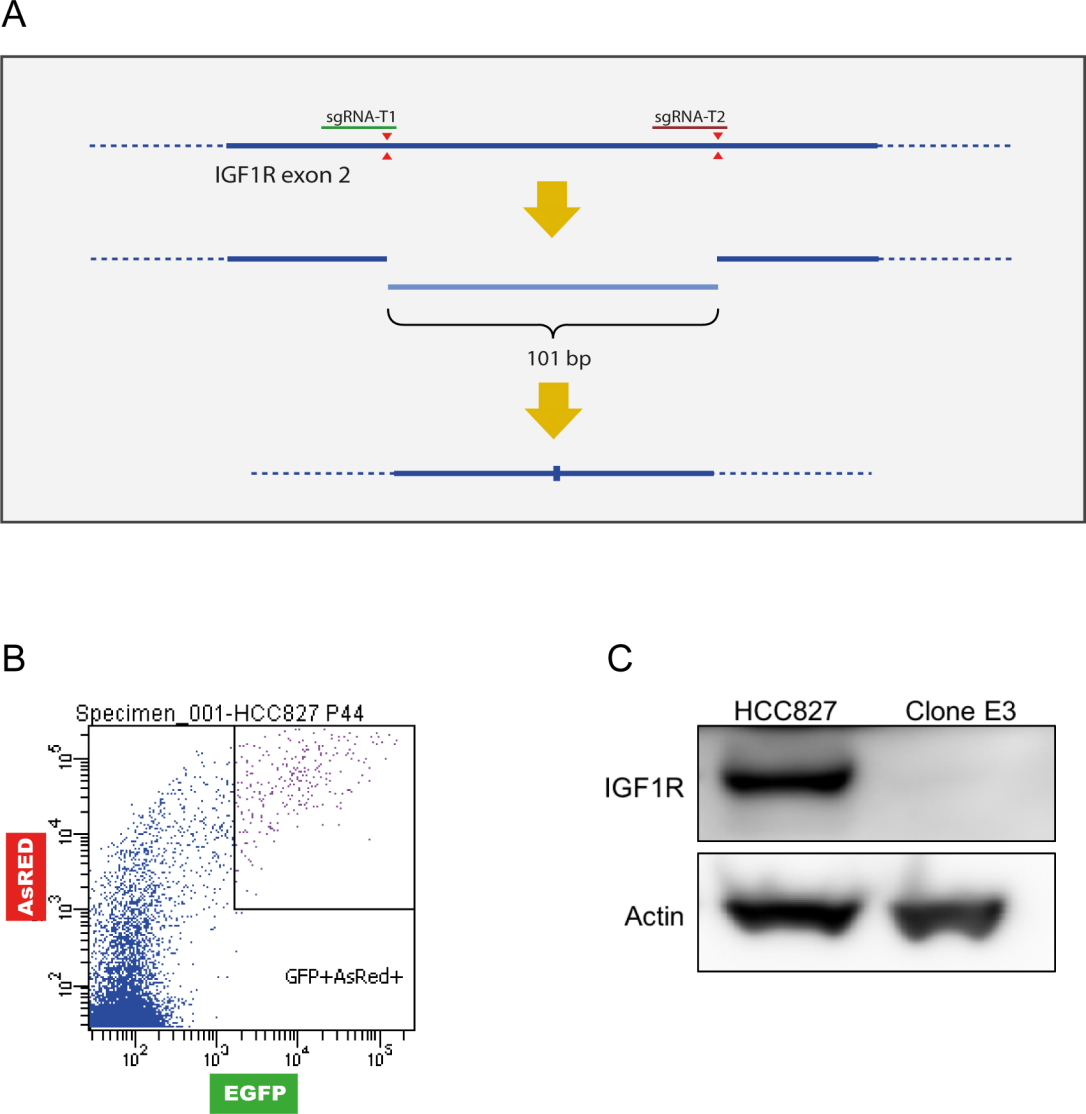
Generation of a HCC827(IGF1R−/−) cell line A HCC827 cell line with *IGF1R* knock-out was created by inducing a genetic deletion using CRISPR/Cas9. **(A)** Schematic representation of sgRNA cleavage (red triangles) for introduction of genetic deletion in exon 2 of the *IGF1R* gene (bold line, dotted line represents introns). Cleavage by two sgRNAs can result in blunt end-joining of the two remaining strands or possible introduction of indels during the repair process. **(B)** Gating strategy for single-cell sorting using C-check reporter vector based on high fluorescence intensity for enrichment of transfected cells (high AsRED) and increased likelihood of gene edited cells (high EGFP). **(C)** Western blot analysis of IGF1R protein expression in HCC827 cells and Clone E3 cells. β-actin was used as loading control.

To clarify the involvement of IGF1R in acquired erlotinib resistance in HCC827 cells, we performed a genetic knockout of the gene. The knockout was mediated using a CRISPR/Cas9 plasmid-based workflow with two sgRNAs plasmids and a dual-fluorescent reporter vector, C-Check, described by Zhou *et al*. [29]. The sgRNAs introduce two distinct double stranded breaks within exon 2 of the *IGF1R* gene (common coding exon of all IGF1R isoforms) giving rise to blunt-end ligation of the remaining strands and a deletion of 101 bp (Figure 2A). The transfected cells were single sorted based on high EGFP and AsRED fluorescence for effective selection of transfected and potentially gene-edited cells (Figure 2B and Supplementary Figure 2A). Five single-cell derived clones expanded successfully, and PCR screening for genetic deletion was performed with primers flanking the targeted region (Supplementary Figure 2B). The genetic deletion was detected by gel electrophoresis as the presence of an approx. 200 bp band (Supplementary Figure 2C). One clone (Clone E3) demonstrated a 100 bp out-of-frame deletion on both alleles (Supplementary Figure 2D) resulting in complete loss of IGF1R protein (Figure 2C). This clone was selected for further analyses and abbreviated HCC827(IGF1R−/−). Sanger sequencing of four potential off-target sites per sgRNA revealed no off-target events in HCC827(IGF1R−/−) (Supplementary Figure 2E).

### HCC827(IGF1R−/−) acquires erlotinib resistance through *MET*-amplification

**Figure 3:**
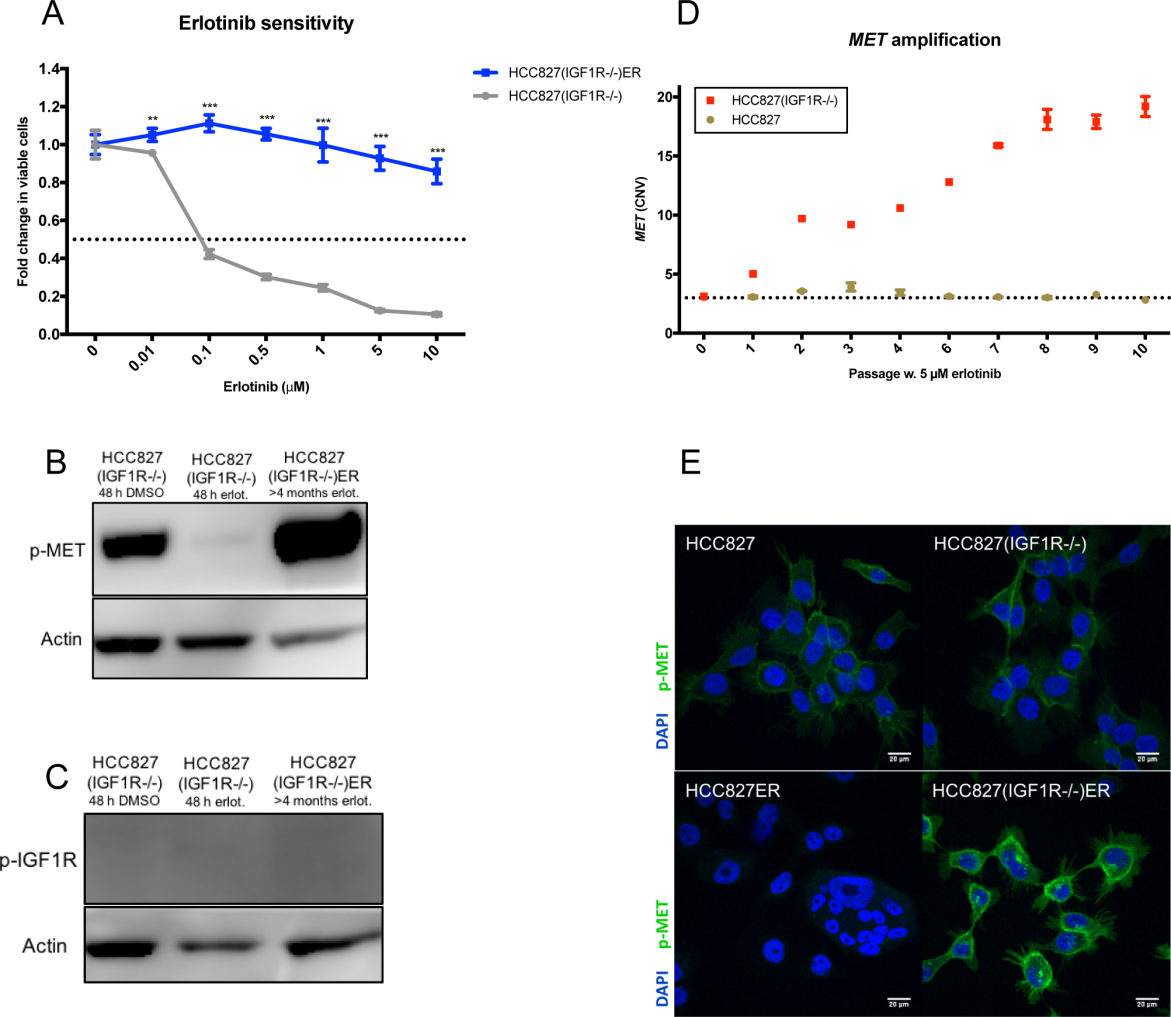
Depletion of IGF1R leads to different bypass-signaling mechanisms during acquired TKI resistance **(A)** Viability in response to treatment with increasing concentrations of erlotinib determined by MTS assay for HCC827(IGF1R−/−) and HCC827(IGF1R−/−)ER. Parental and erlotinib-resistant cells were treated with indicated concentrations of erlotinib for 72 hours before incubation with MTS solution. All erlotinib dilutions (0-10 μM) were corrected to contain equal amounts of DMSO. The dotted line indicates 0.5-fold change in number of viable cells. The number of viable cells was determined relatively to erlotinib-untreated controls, and fold change in viable cells is plotted as mean ±SD. Significance is calculated for each concentration between parental and resistant cell lines. (*p<0.05, **p<0.01, ***p<0.001). **(B and C)** Western blot analysis of **(B)** phosphorylated MET (p-MET) and **(C)** phosphorylated IGF1R (p-IGF1R) protein expression in HCC827(IGF1R−/−) and HCC827(IGF1R−/−)ER. Parental cells were treated with 48 h of DMSO or 5 μM erlotinib prior to protein harvest. β-actin was used as loading control. **(D)** *MET*-amplification was detected by CNV assay using digital droplet PCR in progressive samples obtained through-out resistance development for HCC827 and HCC827(IGF1R−/−). Passage 0 reflects parental cells and passage 10 reflects cells with acquired resistance. The dotted line indicates CNV in parental cells. **(E)** Immunofluorescence staining of phosphorylated MET protein expression (p-MET, green) in HCC827, HCC827ER, HCC8278(IGF1R−/−), HCC827(IGF1R−/−)ER. Cell nuclei are visualized by DAPI (blue). Images are representative for the whole slide, and were captured with fixed settings. Size-bar = 20 μm.

Acquired resistance to erlotinib was induced in HCC827(IGF1R−/−) cells by the same protocol used to generate HCC827ER. After 4 months of 5 μM erlotinib exposure, HCC827(IGF1R−/−)ER were no longer responsive to erlotinib concentrations up to 10 μM (Figure 3A). Next, we investigated resistance mechanisms in the absence of IGF1R by studying mutational and signaling changes between HCC827(IGF1R−/−) and HCC827(IGF1R−/−)ER. HCC827(IGF1R−/−)ER cells were found to retain the original exon19 deletion, and no emerging secondary mutations were detected in the *EGFR* gene (Supplementary Figure 1A). Receptor phosphorylation screening revealed reduced phosphorylation of EGFR and no hyperactivation of any other RTKs, but demonstrated a sustained MET receptor phosphorylation (Supplementary Figure 1C). Western blot analysis further confirmed MET receptor hyperactivation (Figure 3B and Supplementary Figure 3A). Additionally, IGF1R was not found to be activated in HCC827(IGF1R−/−)ER, as observed in HCC827ER, in line with the absence of functional IGF1R protein (Figure 3C and Supplementary Figure 3A). We investigated *MET* copy-number variations throughout the course of resistance development, as this is a well-known erlotinib resistance mechanism. *MET* copy numbers were found to increase during resistance development for HCC827(IGF1R−/−), but remained at basal level during resistance development for HCC827 (Figure 3D). Immunofluorescence staining of phosphorylated MET showed a homogeneous activation of MET receptor in HCC827(IGF1R−/−)ER cells at the cellular level (Figure 3E). In contrast, MET-activation was not observed for HCC827ER cells (Figure 3E).

### Functional dependency of MET and IGF1R activation in HCC827ER and HCC827(IGF1R−/−)ER

**Figure 4:**
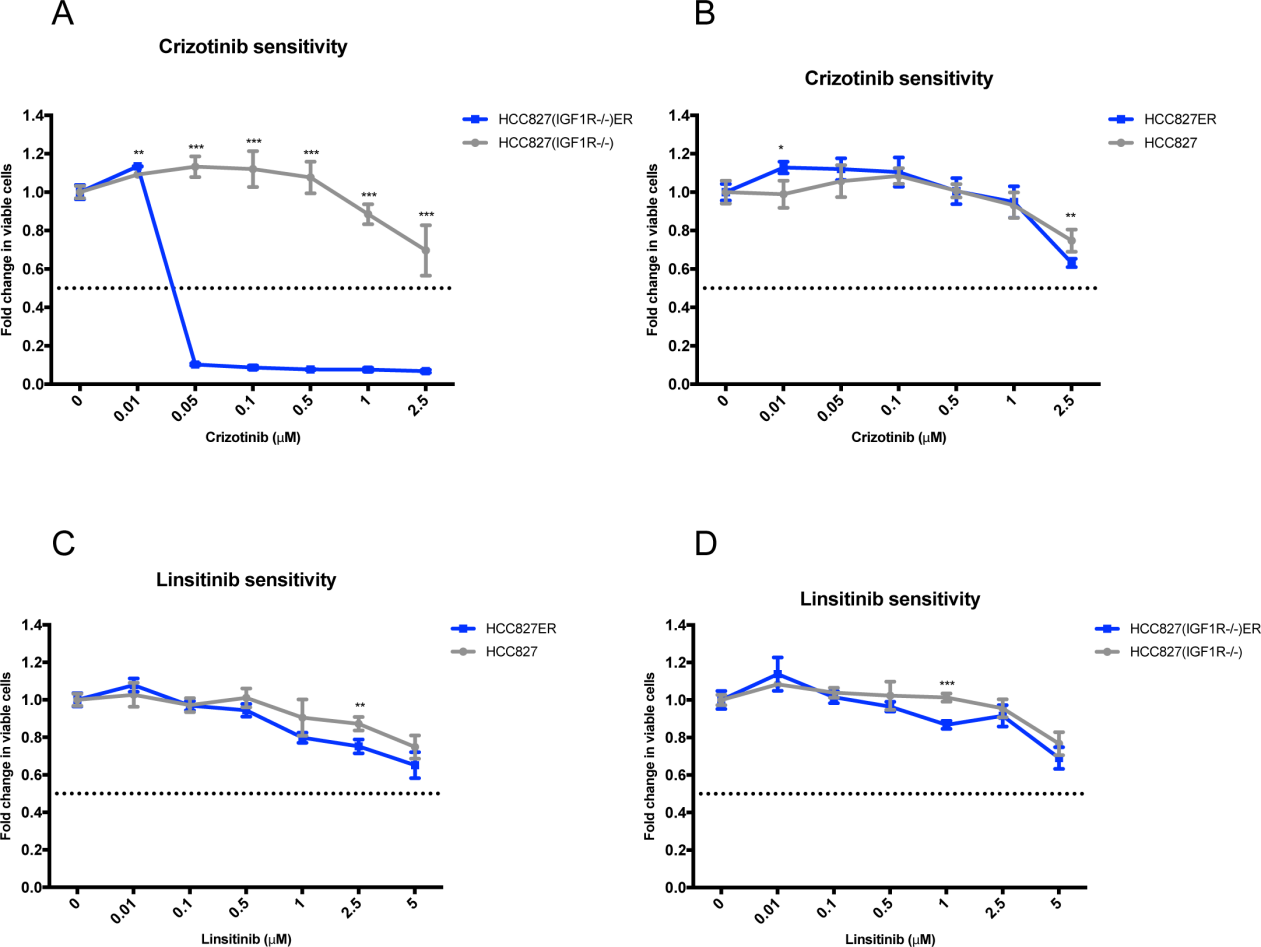
Functional dependency on receptor hyperactivation after acquired resistance Viability response upon 72 h inhibition of the hyper-phosphorylated receptors for each resistant cell line determined by MTS assay. Resistant cells (blue) were additionally grown in 5 μM erlotinib, and parental cells (grey) were grown with equal DMSO load. All linsitinib (0-5 μM) and crizotinib (0-2.5 μM) dilutions were corrected to contain equal amounts of DMSO. The dotted line indicates a 0.5-fold change in number of viable cells. The number of viable cells was determined relatively to untreated controls, and fold change is plotted as mean ±SD. Significance is calculated for each concentration between parental and resistant cell lines (*p<0.05, **p<0.01, ***p<0.001). MET dependency in **(A)** HCC827(IGF1R−/−) and HCC827(IGF1R−/−)ER and in **(B)** HCC827 and HCC827ER assessed as response to increasing doses of crizotinib (MET inhibitor). IGF1R dependency in **(C)** HCC827 and HCC827ER and in **(D)** HCC827(IGF1R−/−) and HCC827(IGF1R−/−)ER assessed as response to increasing doses of linsitinib.

Inhibitor sensitivity assays confirmed that the MET-activated HCC827(IGF1R−/−)ER cell population was indeed dependent on MET signaling as even low concentrations of crizotinib (MET inhibitor) decreased the viability of HCC827(IGF1R−/−)ER cells (Figure 4A). HCC827ER showed no sensitivity to MET inhibition (Figure 4B) in line with the absence of MET activation. HCC827ER cells were not found to have an increased dependency on IGF1R signaling, compared to HCC827 cells, despite IGF1R hyperactivation, as IGF1R inhibition by linsitinib did not significantly decrease cell viability (Figure 4C). HCC827(IGF1R−/−)ER showed no sensitivity to linsitinib (Figure 4D).

### Examination for EMT features in HCC827ER and HCC827(IGF1R−/−)ER

**Figure 5:**
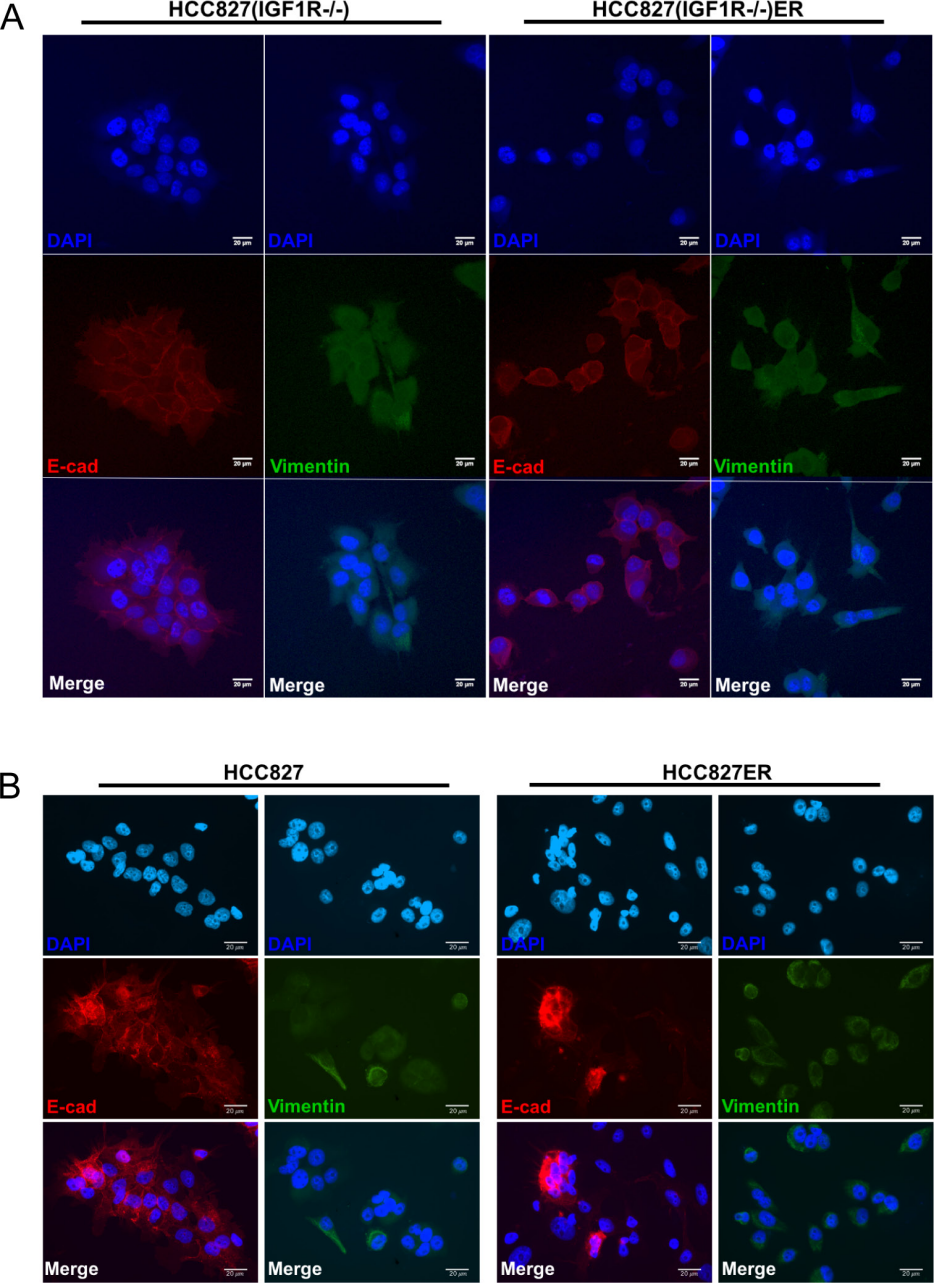
Acquirement and absence of EMT features in HCC827ER and HCC827(IGF1R−/−)ER cells, respectively Immunofluorescence staining of E-cadherin (red) and vimentin (green) protein expression in **(A)** HCC827(IGF1R−/−) and HCC827(IGF1R−/−)ER cells and **(B)** HCC827 and HCC827ER cells. Cell nuclei are visualized by DAPI (blue). Images are representative for the whole slide and were captured with fixed settings for each parental/resistant cell line. Size-bar = 20 μm.

An EMT phenotypic shift is often associated with acquired TKI resistance. HCC827ER and HCC827(IGF1R−/−)ER cells were immunostained for EMT markers (Figure 5). High E-cadherin (*CHD1)* protein expression is a general hallmark of an epithelial phenotype, whereas high vimentin (*VIM*) and high N-cadherin (*CDH2*) protein expression are characteristic of a mesenchymal phenotype. HCC827(IGF1R−/−)ER lacked characteristics of EMT, as the cells in general maintained high E-cadherin expression and did not increase the basal expression of vimentin present in HCC827(IGF1R−/−) cells (Figure 5A). HCC827 was in general highly E-cadherin positive, but HCC827ER demonstrated a heterogeneous expression pattern of E-cadherin, consisting of widespread E-cadherin-negative cells and patches of E-cadherin-positive cells (Figure 5B). Vimentin expression showed no obvious increase in HCC827ER cells, as the cells already demonstrate a relatively high vimentin expression (Figure 5B). Western blot analyses verified the cadherin switch from E-cadherin til N-cadherin in HCC827ER cells (Supplementary Figure 3B). Thus, HCC827ER cells demonstrated a heterogeneously acquisition of EMT features, characteristic for only a subpopulation of the resistant cells.

**Figure 6:**
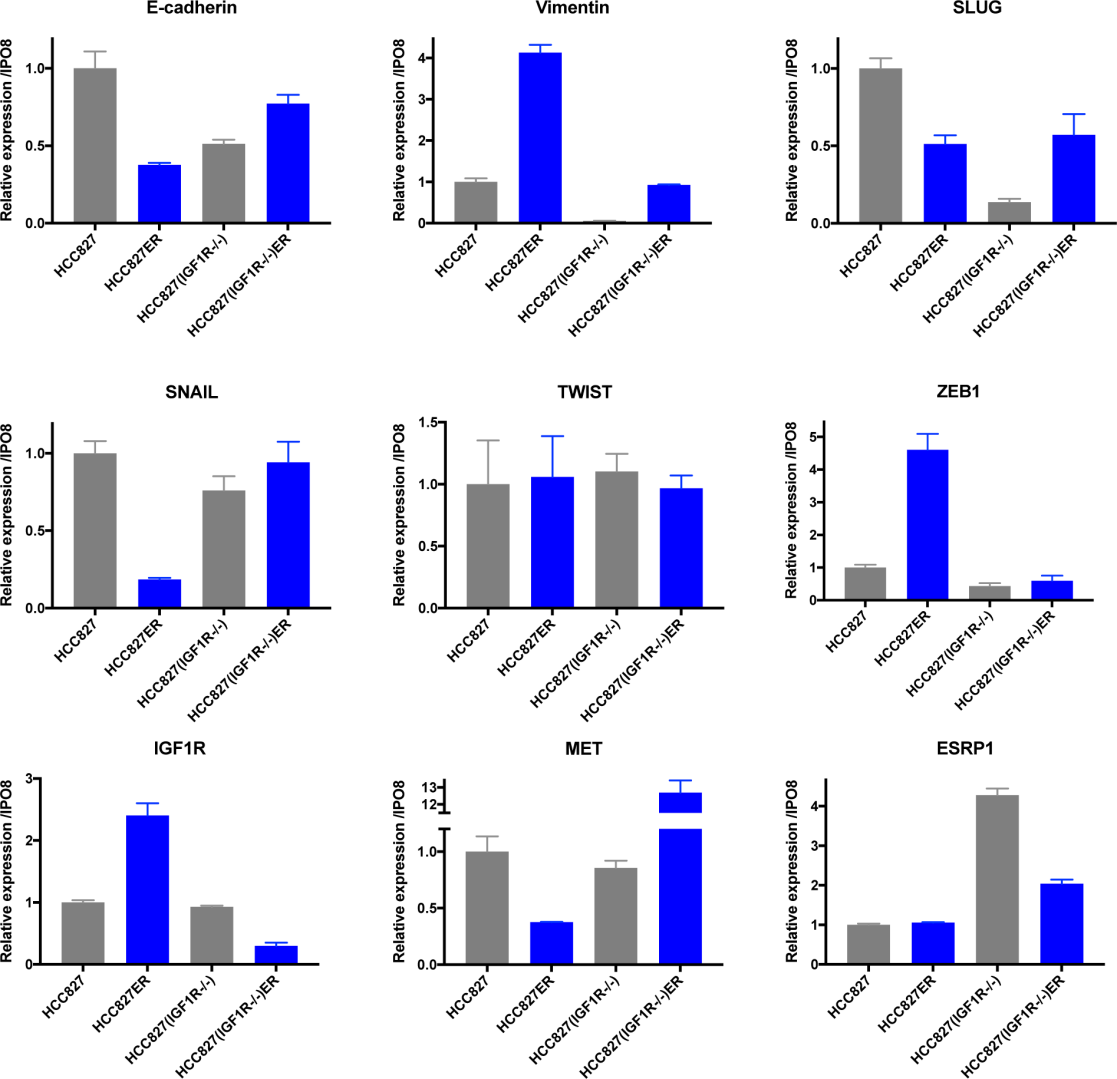
EMT marker expression after acquired erlotinib resistance mRNA expression levels were assessed by qPCR analysis in parental (grey) and resistant (blue) cell populations. Gene expression of each target was normalized to *IPO8* and expression level was presented relatively to HCC827 for HCC827ER, HCC827(IGF1R−/−), and HCC827(IGF1R−/−)ER. Expression levels are based on one biological sample and illustrated as mean ± SD.

The mRNA expression signature of EMT was further investigated in the parental and resistant cell populations. Surprisingly, we found the low expression level of several mesenchymal markers (vimentin*, SLUG, ZEB1*) to be reduced in HCC827(IGF1R−/−) compared to HCC827 (Figure 6, grey columns). Additionally, the gene expression of the epithelial marker, *ESRP1*, was increased fourfold in HCC827(IGF1R−/−), implying HCC827(IGF1R−/−) to have a more epithelial-like gene expression signature compared to HCC827 (Figure 6, grey columns). The HCC827ER cells were characterized by a decrease in E-cadherin gene expression and an increase in vimentin gene expression assisted by an increased expression of the EMT marker *ZEB1* (Figure 6, blue versus grey columns). Hence, the gene expression analysis of HCC827ER confirmed the presence of EMT features, which can be assigned to a subpopulation of the HCC827ER cells. HCC827(IGF1R−/−)ER showed, compared to HCC827(IGF1R−/−), no distinct changes in E-cadherin expression, but increased expression of vimentin and *SLUG* (Figure 6, blue versus grey columns). This increase in vimentin expression was, however, not observed on protein level by immunostaining (Figure 5A), which could be explained by a generally low level of mRNA expression (Figure 6).

### Examination of the initial response to erlotinib

To establish a more detailed pattern of gene expression during erlotinib resistance, we measured mRNA levels at each passage throughout resistance development in both HCC827 and HCC827(IGF1R−/−) cells (Supplementary Figure 4). As previously described, *IGF1R* expression increased early during HCC827 resistance development. This was not observed in HCC827(IGF1R−/−) cells, pointing to an auto regulatory loop for IGF1R (Supplementary Figure 4). We next examined several mesenchymal EMT markers (Supplementary Figure 4). *TWIST* was the only marker that showed a trend in expression changes with an early upregulation in HCC827, which progressively decreased throughout the subsequent passages. No expression changes were observed in HCC827(IGF1R−/−).

**Figure 7:**
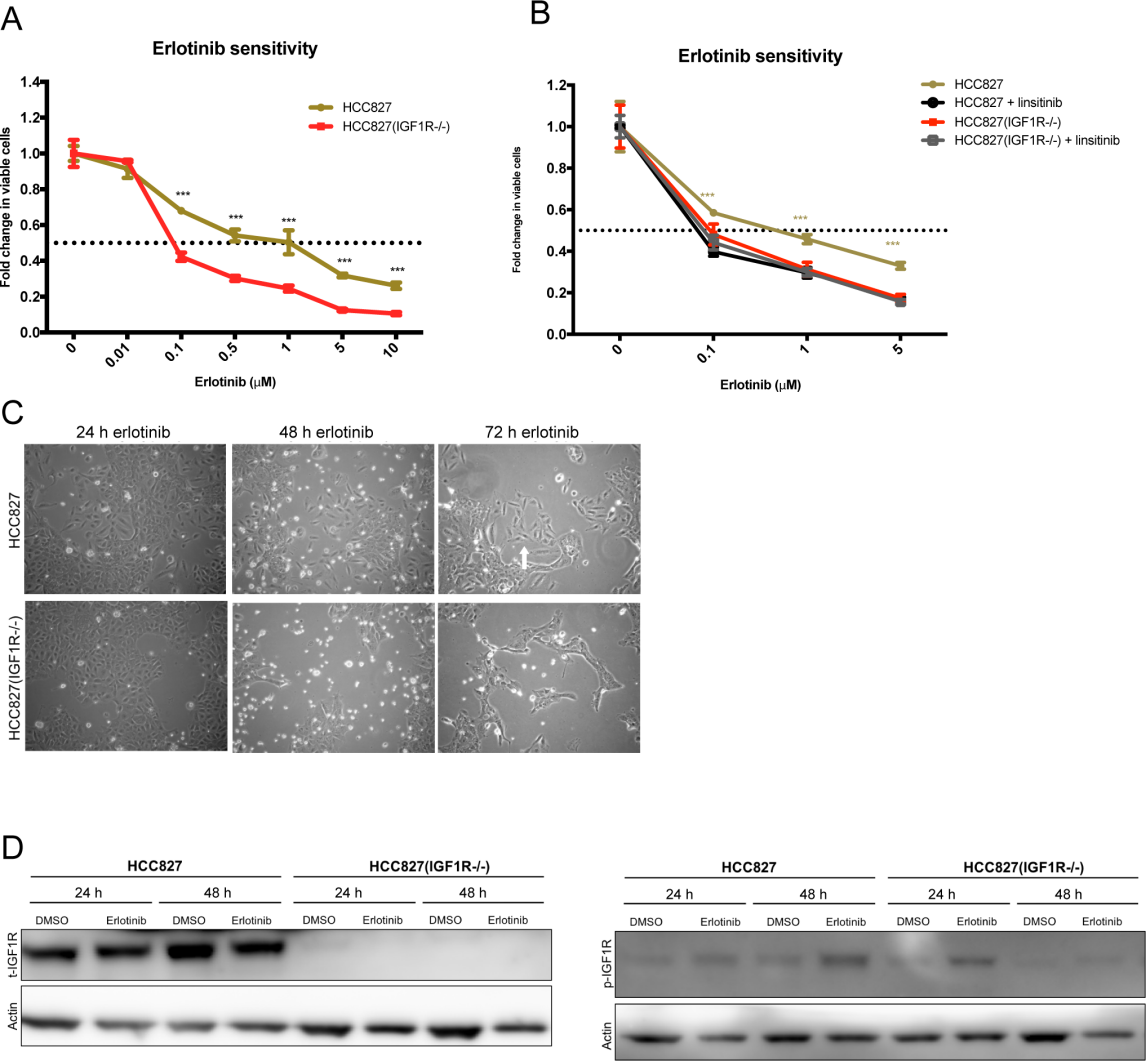
IGF1R activation in the initial response to erlotinib **(A)** Erlotinib sensitivity in parental HCC827 and HCC827(IGF1R−/−) measured by MTS as viability response to increasing concentrations of erlotinib. The figure shows combined data from Figure 1A and 3A (grey curves). Significance is calculated between the two parental cell lines. (*p<0.05, **p<0.01, ***p<0.001). **(B)** Erlotinib sensitivity in parental HCC827 and HCC827(IGF1R−/−) measured by MTS as viability response to increasing erlotinib concentrations alone (beige and red) or with concurrent inhibition of IGF1R using 2.5 μM linsitinib (black and grey). Significance is calculated between erlotinib versus erlotinib/linsitinib exposure for each cell line. (*p<0.05, **p<0.01, ***p<0.001). (A+B) Cells were treated with indicated concentrations of erlotinib for 72 hours before incubation with MTS solution. All dilutions were corrected to contain equal amounts of DMSO. The dotted line indicates 0.5-fold change in number of viable cells. The number of viable cells was determined relatively to erlotinib-untreated controls, and fold change in viable cells is plotted as mean ±SD. **(C)** Phase-contrast images of cell morphology in HCC827 and HCC827(IGF1R−/−) cells after transient exposure to 5 μM erlotinib. Images were captured at 10X magnification after 24 h, 48 h, and 72 h of treatment. **(D)** Western blot analysis of total IGF1R (t-IGF1R) and phosphorylated IGF1R (p-IGF1R) protein expression after 24 h and 48 h of exposure to 5 μM erlotinib. Note that the phospho-IGF1R antibody cross-reacts with phosphorylated insulin-receptor. Control cells were treated with similar DMSO load. β-actin was used as loading control.

Due to the lack of dependency on IGF1R signaling in HCC827ER (Figure 4C), IGF1R was speculated to exert its role during the initial response to erlotinib exposure. Therefore, we subjected parental cells to transient erlotinib exposure for 24-72 hours. During the first 72 hours, HCC827 and HCC827(IGF1R−/−) cells responded differently to erlotinib exposure. HCC827(IGF1R−/−) cells were significantly more sensitive to erlotinib than HCC827 (Figure 7A). Interestingly, erlotinib sensitivity was increased in HCC827 upon concurrent inhibition of IGF1R for 72 hours using the IGF1R-TKI, linsitinib (Figure 7B). Moreover, during erlotinib exposure a fraction of the HCC827 cells displayed a different morphological appearance with elongated and widespread cells, compared to the HCC827(IGF1R−/−) cells being tightly adhered and cuboidal-shaped (Figure 7C). Receptor phosphorylation screening revealed no clear bypass-signaling in HCC827 and HCC827(IGF1R−/−) erlotinib exposed cells, but EGFR and MET-activation was pronouncedly decreased after 48 hours of erlotinib exposure (Supplementary Figure 5A). This was accompanied by a decrease in *MET* gene expression (Supplementary Figure 5C). Surprisingly, given that MET activation was the resistance endpoint of HCC827(IGF1R−/−) cells, we observed a more pronounced decrease in activated MET in HCC827(IGF1R−/−) compared to HCC827 cells following 48h of erlotinib exposure (Supplementary Figure 5A and 5B). *IGF1R* mRNA expression was increased in both HCC827(IGF1R−/−) and HCC827 cells upon erlotinib exposure for 24h and 48h (Supplementary Figure 5C). Western blot examination of IGF1R protein showed no clear trend following 5 μM erlotinib exposure for 24h and 48 h (Figure 7D). Western blot analyses of activated IGF1R in HCC827 cells after erlotinib exposure showed only weak signals and given that bands with similar size were observed in a parallel analysis of HCC827(IGF1R−/−) cells together with no detection of total IGF1R protein, we cannot conclude if this is due to cross reactivity against p-IGF1R related epitopes, such as phosphorylated insulin receptor (Figure 7D). Gene expression results for various EMT markers showed a similar response in HCC827(IGF1R−/−) and HCC827 cells after 48 hours of erlotinib exposure (Supplementary Figure 5C) with similar induction of mesenchymal EMT markers *SLUG*, *TWIST*, and vimentin (*VIM*) but at the same time *SNAIL* repression (Supplementary Figure 5).

## DISCUSSION

Pharmacological inhibition or siRNAs have previously been employed for downregulating RTKs in relation to TKI resistance in NSCLC cell lines [8, 22, 28, 30–33]. The efficiency of functional knockdown by these methods can, however, be questioned, and it is likely that some functional receptor is still present in the cells. To our knowledge, our study is the first developing TKI resistant cells from a functionally depleted genetic knockout cell line. In this way, we ensure that no functional protein of interest is present. Furthermore, most other studies have investigated the role of specific receptors following acquired resistance. Here, we enlighten mechanisms of acquired resistance in the absence of a specific receptor, namely IGF1R, in the genetic background of the NSCLC cell line HCC827. By functionally removing IGF1R through genetic deletion in HCC827 cells, the *IGF1R* knockout cell line HCC827(IGF1R−/−) was generated. Subsequently we established HCC827 and HCC827(IGF1R−/−) erlotinib-resistant sublines, HCC827ER and HCC827(IGF1R−/−), respectively, by continuous high-dose erlotinib exposure. Under these conditions HCC827ER cells hyperactivated IGF1R, but showed no functional dependence on IGF1R signaling compared to parental HCC827. Furthermore, heterogeneous display of EMT features was identified among the HCC827ER cells, indicating that a subpopulation of cells underwent EMT. Interestingly, HCC827(IGF1R−/−)ER gained erlotinib resistance by a different mechanism, namely *MET*-amplification with resulting MET hyperactivation. In addition, HCC827(IGF1R−/−)ER cells did not gain EMT features like HCC827ER cells. Previous studies have suggested a role for IGF1R in initiating EMT [8, 28]. As IGF1R was not necessary for maintaining an already gained erlotinib resistance in HCC827ER cells, we speculated whether IGF1R signaling was important for initiating EMT during the early steps of resistance development. Therefore, we studied the initial response to erlotinib and indeed identified an early increase in *IGF1R* and *TWIST* gene expression in HCC827 cells. In addition, HCC827 and HCC827(IGF1R−/−) cells responded differently to transient erlotinib exposure (5 μM up to 72 hours), with a subpopulation of HCC827 cells exerting more migratory growth and spindle-shaped morphology. HCC827(IGF1R−/−) cells demonstrated higher erlotinib sensitivity than HCC827, which we were able to induce in HCC827 cells by concurrently exposing the cells to erlotinib and an IGF1R inhibitor, linsitinib. Altogether, these findings show that the HCC827(IGF1R−/−) cells are able to develop resistance to high-dose erlotinib without functional IGF1R. However, they do so in a homogenous manner utilizing bypass-signaling via MET hyperactivation through *MET*-amplification. In contrast, parental HCC827 cells develop resistance to high-dose erlotinib through a process characterized by IGF1R hyperactivation and heterogeneously acquirement of EMT features, and no evidence of MET hyperactivation. Experimental observations have pointed *EGFR*-mutated cell lines to exhibit “desire” to utilize certain resistance mechanisms under a given TKI selection pressure [10, 34–39]. Previous studies have shown HCC827 to have a “desire” to establish resistance through *MET*-amplification [10, 16, 32, 33, 40, 41]. Yet, all these studies have applied a protocol using low or stepwise escalating doses of EGFR-inhibitors starting from 5-10 nM [10, 16, 32, 33, 41]. A concern regarding method of choice was pointed by Shien *et al*. as they developed HCC827 gefitinib resistant cells by both escalating and high dose protocols, which resulted in either resistance through *MET*-amplification (escalating doses) or acquirement of EMT and stem cell features (high-dose) [10]. Multiple high-dose *in vitro* models of acquired resistance (minimum 1 μM) have presented EMT features [8, 10, 28, 42]. Together, these studies indicate that the method of choice might direct the “desired” resistance mechanisms. Our results and the recent literature imply that when cells are pressured *in vitro* by low or stepwise-escalating doses of TKIs, specific resistance mechanisms such as *MET*-amplification tend to emerge. When cells are pressured by high drug concentrations, resistance mechanisms such as EMT seem more likely to occur. Interestingly, by removing some of the available tools within the cell, here demonstrated by functional IGF1R depletion, the balance between “desired” resistance mechanisms are shifted. A model illustrating this is presented in Supplementary Figure 6. HCC827ER cells heterogeneously displayed EMT features, pointing that only a subpopulation of cells underwent EMT. The driving forces of resistance for the non-EMT HCC827ER subpopulation remain elusive, but immunofluorescence analyses excluded MET hyperactivation. The observed heterogeneity stresses another complexity in resistance development; the emergence of parallel resistance mechanisms [43]. In HCC827(IGF1R−/−) cells we observed homogeneous emergence of MET hyperactivation. We speculate that the functional removal of IGF1R limits the mechanisms of choice in the cells, and in this context *MET*-amplified clones hold the greatest potential to outmatch other emerging resistant clones. IGF1R stimulation or silencing has additionally been observed to direct the cells towards or away from a mesenchymal phenotype [44, 45], and E-cadherin positive cells have been found to be more sensitive to erlotinib compared to E-cadherin negative cells [8, 46]. This is consistent with our observation that HCC827(IGF1R−/−) cells showed increased sensitivity to erlotinib and demonstrated a reduced basal gene expression level of several EMT factors compared to HCC827 cells. HCC827(IGF1R−/−) cells might be comprehended as having a “super-epithelial”-like gene expression signature, compared to HCC827 cells, and thus a superior threshold for undergoing EMT. Limitations of our study is the restriction to a single NSCLC genetic background only comparing parental HCC827 cells versus HCC827(IGF1R−/−) cells under resistance development. Our main focus was on resistance endpoints, and hence, emergence of *MET*-amplified subclones might have been present during the development of erlotinib resistance also in parental HCC827 cells. Nevertheless, cells utilizing other pathways than the identified must hold inferior potential in competition with the mechanisms demonstrated at resistance endpoint. Comprehended in the light of literature, we here provide an interesting result to the observed systematical shift between the “desired” mechanisms using high dose versus escalating dose protocols. Ultimately, our observations indicate that the resistance development is a flexible process, and that the cells find other ways of developing resistance when their “desired” tools are lacking.

Directing the “desired” resistance mechanism by pharmacological inhibition has successfully been proven *in vitro*. Sesumi *et al*. [47] and Soucheray *et al*. [48] directed NSCLC HCC4006 cells to EGFR T790M-driven resistance to gefitinib and erlotinib by pharmacologically inhibiting the otherwise recurrently observed EMT resistance mechanism. Using a stepwise-escalating dose protocol, Suda *et al*. likewise directed HCC827 to acquire erlotinib resistance through T790M EGFR-mutation by co-treatment with a MET inhibitor [41]. Co-treatment strategies are of clear clinical relevance for overcoming resistance, when considering both the ductility of resistance development and the emergence of parallel resistance mechanisms. However, the toxicities regarding co-treatment appear to be of great concern. Clinical trials have investigated the combinatorial treatment using IGF1R inhibitors in addition to cytotoxic chemotherapy or EGFR inhibitors [49]. A recently completed phase II trial has reported inferior outcome for *EGFR*-mutated NSCLC patients co-treated with erlotinib and linsitinib (IGF1R-directed TKI, OSI-906) compared to erlotinib alone [50]. This could be partly due to discontinued and earlier termination of erlotinib exposure in the co-treated patient group, as these patients experienced increased adverse effects. Thus, these clinical studies highlight the complexity of simultaneously targeting different signaling pathways and the need for understanding the mechanistic and molecular interplay in NSCLC TKI resistance development.

In conclusion, our results show that IGF1R signaling under the given experimental conditions and NSCLC genetic background dictates the functional endpoint mechanism for TKI resistance. Manipulating this regulatory role of IGF1R can force the functional endpoint mechanism for TKI resistance in a defined and targetable direction here illustrated by the observed *MET*-amplification. The number of clinically verified TKIs keeps expanding, paving the way for co-treatment approaches, and knowledge of the ductility of resistance mechanisms, both clinically and experimentally, will have obvious clinical relevance for guiding treatment strategies.

## MATERIALS AND METHODS

### Cell culture and reagents

HCC827 (ATCC® CRL-2868) was purchased from ATCC. Cells were cultured in RPMI-1640 media supplemented with L-glutamine (BioWhittaker, Lonza), 10 % FCS, 1 % pen/strep, 1 mM sodium pyruvate, 10 mM Hepes (pH 7) and 2.5 mg/L amphotericin B (Sigma Aldrich) (complete medium) and grown in a humidified incubator with 5 % CO_2_ at 37°C. Tyrosine kinase inhibitors erlotinib, linsitinib and crizotinib were obtained through Selleckhem as 10 mM stock solutions dissolved in DMSO and stored at −80°C.

### Generation of HCC827(IGF1R−/−) cell line

#### Plasmids

The HCC827(IGF1R−/−) cell line was created by introducing two double stranded breaks with 101 base pair (bp) distance in the *IGF1R* gene in HCC827 cells, resulting in a deletion within a common coding exon in *IGF1R*. Generation of the CRISPR/Cas9 knock-out system comprising sgRNA-plasmids targeting exon 2 of *IGF1R*, the C-Check reporter vector containing the *IGF1R* target region and validation of plasmids and cleavage efficiency are described in detail by Zhou et al [29].

#### Transient transfection and fluorescence-activated cell sorting

Approximately 1.5×10^6^ HCC827 cells were seeded into a 10 mm dish 48 h prior to transient transfection. Cells were transfected using X-tremeGENE 9 DNA Transfection Reagent (Roche) according to manufacturer's protocol. A total of 8.1 μg plasmid-DNA was used for transfection equally divided between the three plasmids: sgRNA-T1, sgRNA-T2 and C-Check reporter vector. Seventy-two hours post transfection the cells were harvested using trypsin, washed twice with 2% FCS in PBS and suspended in 600 μL 2% FCS in PBS. Fluorescence-Activated Cell Sorting (FACS) were performed using a four-laser FACSAria III cell sorter, and the cells were single-sorted based on high fluorescence intensity of AsRED and EGFP into 100 μL medium supplemented with 5 mM HEPES. Clonal expansion was performed in complete medium, which was changed every 3-4 days. After 4 weeks 5 colonies were screened for genomic knock-out (Supplementary Table 1).

### RNA purification and cDNA synthesis

Cells for RNA purification were harvested by scraping directly from the culture plates, centrifuged for 3 minutes at 1800 rpm and pellets stored at −20°C until purification.

Total RNA was obtained from cell pellets using QIAcube (Qiagen) with either RNeasy Mini Kit (Qiagen) or RNeasy Micro Kit (QIAGEN) according to the manufacturer's protocols. RNA concentrations were measured using a Nanodrop 2000 Spectrophotometer (Thermo Scientific). cDNA was synthesized from 0.1 μg of total RNA in a 20 μL reaction mixture consisting of PCR buffer(1X), MgCl_2_ (6.25 mM), RNase Inhibitor (20 units), MuLV Reverse Transcriptase (50 units) (Applied Biosystems), 1 mM of each dNTP (VWR) and Oligo(dT)_16_ (2.5 μM, DNA Technology). Reverse transcription was performed on the GeneAmp®PCR system 9700 (Applied Biosystems) using the following protocol: 42°C for 30 min, 99°C for 5 min and cooled to 4°C. cDNA was stored at −20°C and RNA at −80°C.

### Quantitative real-time PCR (qPCR)

Gene expression was investigated using quantitative real-time PCR (qPCR) on a Lightcycler®480 instrument (Roche). The reaction mixture consisted of Lightcycler®480 SYBR Green I Master (1X), forward primer (0.25 μM), reverse primer (0.25 μM) with 1 μL cDNA input in a total volume of 10 μL. All primers were ordered from Eurofins Genomics and are listed with assay-specific information in Supplementary Table 2. The reactions were conducted in 96-well plates using the protocol: 10 min of heating at 95°C followed by 50 cycles of denaturation at 95°C for 10 sec., annealing at assay-specific T_A_ for 20 sec. and finally elongation at 72°C for 5 sec. Melting curves were obtained by subsequent cooling to 40°C at a transition rate of −2.2°C/sec to verify homogeneous product amplification. mRNA concentrations were calculated using the standard curve method. Several reference gene candidates were investigated in all experimental set-ups using the web-accessible Normfinder Software [51], and *IPO8* were found to be the most suitable gene for all individual experiments. Gene expression analyses of progressive samples obtained during resistance development are presented as mean ± SD, as this is based on one biological sample.

### DNA purification

Cells for DNA purification were harvested by scraping directly from culture plates, centrifuged for 3 minutes at 1800 rpm and pellets stored at −20°C until purification. DNA was purified using QIAcube (Qiagen) with the QIAamp DNA Blood Mini Kit (50) according to manufacturer's protocols. DNA concentrations were measured using a Nanodrop 2000 Spectrophotometer (Thermo Fisher Scientific). DNA was stored at −80°C.

### Genetic analysis

Copy-number variations of *MET* were assessed using the PrimePCR™ ddPCR™ Copy Number Assay (MET, human) on the QX200™ DropletReader instrument (BioRad) according to the manufacturer's protocol. EGFR allele-specific PCR was performed on a Cobas Z 480 instrument (ROCHE) using the Cobas® EGFR mutation Testv2 kit (Roche) according to manufacturer's protocol, providing EGFR allele frequency in arbitrary units.

### Protein harvesting

Protein harvest from 6-well plates: Protein was obtained by adding 100-200 μL Lysis buffer 17 (R&D Systems) with 10 μg/mL protease inhibitors aprotinin, leupeptin, and pepstatin directly to cells in 6-well plates. After 30 min of shaking at 4°C, the lysates were spun for 5 min at 14000g at 4°C and pellet discarded. Protein concentrations were measured on a Qubit®2.0 Fluorometer using Qubit®Protein Assay Kit (Thermo Fisher Scientific) following the manufacturer's instructions. Protein samples were stored at −80°C. Protein harvest from T75 flasks: Cells were scraped and the pellet were lysed with 100-150 μL lysisbuffer (50 mM Tris HCL pH 7.8; 5 mM EDTA 0.5M pH 8.0; 1 mM DTT; 10 μg/mL; 1 mg/mL soya bean trypsin inhibitor; 0.5 % triton x-100). Samples were placed on dry ice for 3 min., 37°C for 3 min. and then vortexed three times. Samples were incubated for 30 min. on ice before spun at 10000 rpm for 10 min at 6°C. Protein concentration were measured in the supernatant using Bradford.

### Western blot analysis

Equal amounts of protein samples, either 20 μg or 40 μg, were mixed with NuPAGE LDS Sample Buffer (1X), NuPAGE Reducing Agent (1X) and UV-H_2_O and electrophoresed on a NuPAGE 4-12% Bis-Tris Gel. The gel was blotted for 7 min at 20 V onto an iBLOT Transfer Stack PVDF membrane using the iBlot® Gel Transfer Device (all products from Novex™, Thermo Fisher Scientific). The membrane was divided according to targets and blocked for 1 h with either 5% BSA or 5% skimmed milk according to primary antibody. Primary antibodies were diluted with blocking buffer and incubated overnight with rotation at 4°C. The membranes were washed with 1X TBS with 0.1 % TWEEN-20 before incubation with HP-conjugated secondary antibody for 1-2 h at room temperature with rotation. Protein targets were detected with SuperSignal WestDura Extended Duration Substrate ECL (Thermo Fisher Scientific) and membranes developed using an ImageQuant LAS 4000 system (GE Healthcare Life Sciences) and IQLAS4000 Control Software. After ECL detection, some membranes were re-used. These were placed in 5 mL stripping buffer (2 % SDS, 62.5 mM Tris-HCL, pH 6.7 in distilled water) mixed with 100 mM β-mercaptoethanol at 55°C with rotation for 30 min. The membranes were subsequently washed 2X with 1X TBS 0.1 % TWEEN-20 for 10 min before ordinary protocol was followed from blocking step. Note that the phospho-IGF1R antibody cross-reacts with phosphorylated insulin-receptor. Antibody information is listed in Supplementary Table 3.

### Immunofluorescence staining

100,000 cells were plated on 0.17 mm thick poly-lysine coated cover slips (Marienfeldt) in a 12-well plate. The next day cells were fixed at approx. 70 % confluency with 4 % paraformaldehyde in PBS for 20 min and permeabilized with 0.5 % Triton X-100 PBS for 10 min. Cover slips were blocked with 1%-BSA PBS for 1-1.5 h RT, and subsequently incubated with primary antibody dissolved in 1%-BSA PBS for 1 h followed by incubation with secondary antibody dissolved in 1%-BSA PBS for 30-60 min at RT. Cell nuclei were stained with DAPI (Sigma Aldrich) for 2-5 min. Cells were washed thoroughly with PBS between each step. Cover slips were dipped in demineralized water to prevent crystallization and dried at RT before mounted with Prolong® Gold antifade reagent (Invitrogen) onto glass slides. Pictures were acquired on a ZEISS LSM 710 confocal microscope through a 63X oil immersion objective using a 405 nm diode laser and 488 nm multiline argon laser or on a Zeiss axiovert 200 m microscope with a plan apochromatic 40X objective, a HBO 100 W mercury light source, and a CoolSNAP-HQ cooled CCDcamera (Photometrics) operated by MetaMorph®. All images were obtained with fixed settings for each protein target, and merged using ImageJ. Antibody information is listed in Supplementary Table 4.

### Establishment of erlotinib-resistant cell lines

Erlotinib-resistant cell lines were established by a continuous high-dose approach using 5 μM erlotinib exposures. Parental cells (PAR) were initially grown to approx. 80 % confluency in T75 culture flask before erlotinib exposure (P0). Cells were cultured and passaged (when approx. 90 % confluent) in 5 μM erlotinib until they were able to grow unaffected with erlotinib and then considered resistant. For every passage (P0-P10) half of a flask were scraped and divided into two aliquots, centrifuged for 3 min at 1800 g and pellets stored at −70°C for RNA and DNA purification. The remaining cells were trypsinized, and half were passaged on with erlotinib and half were stored as cell stock. Fresh media was added every 72-96 h. Erlotinib resistance (ER) was reached after 10 passages (approx. 4 months) for both HCC827 and HCC827(IGF1R−/−), denoted HCC827ER and HCC827(IGF1R−/−)ER, respectively. Resistant cells were continuously grown in 5 μM erlotinib for all experiments unless noted.

### Inhibitor assays

Cell viability as response to drug exposure was evaluated using a CellTiter 96® AQ_ueous_ Non-Radioactive Cell Proliferation Assay (Promega). Cells were seeded with a density of 5,000 cells/well in 200 μL media in a 96-well plate (Nunc™) 1 day prior to drug exposure. Cells were treated in 4 replicates with media containing different amounts of the indicated inhibitors. All drug dilutions and controls were adjusted to contain equal amounts of DMSO. After 72 hours of drug exposure, 25 μL MTS mixture were added to each well and left for 2-4 hours incubation. Media were re-suspended in wells and absorbance was measured on 100 μL media at 490 nm (background 690 nm) using a Multiscan GO microplate reader (Thermo Fisher Scientific). Erlotinib-resistant cells (ER) were initially seeded in 5 μM erlotinib, and were grown in 5 μM erlotinib for linsitinib and crizotinib exposure experiments. Inhibitors were tested in concentrations ranging from 0.001-10 μM. For all absorbance's the mean of MTS mixture background is subtracted and the results are presented relatively to control cells (0 μM drug) as mean ± SD based on 4 biological replicates.

### Transient erlotinib exposure

Parental HCC827 and HCC827(IGF1R−/−) cells were treated with 5 μM erlotinib and the control cells with similar DMSO load for 24 hours and 48 hours. Six wells (from 6-well plate) of protein lysate were pooled into one sample for protein expression studies. β-actin was used as loading control. Gene expression studies were performed in biological triplicates and the experiment was conducted twice. Expression of each target gene was normalized to the expression of *IPO8*. Gene expression data represents results from one experiment, illustrated as mean ±SD based on three biological samples.

### Statistical analyses and graphs

Graphs were generated using GraphPad Prism 6 software. Statistical analyses were performed in Prism using the “Multiple t test – one per row”- function, not assuming consistent standard deviation (SD) and with no corrections for multiple comparisons. P-values <0.05 were considered statistical significant.

## SUPPLEMENTARY MATERIALS FIGURES AND TABLES



## Abbreviations

bp: Basepairs
Cas9: CRISPR associated protein 9
CRISPR: Clustered Regularly Interspaced Short Palindromic Repeats
ddPCR: Droplet digital polymerase-chain reaction
DMSO: Dimethyl sulfoxide
EGFR: Epidermal growth factor receptor
EMT: Epithelial-mesenchymal transition
ER: Erlotinib resistant
FACS: Fluorescence-activated cell sorting
FGFR1: Fibroblast Growth factor receptor 1
HD: High-dose
h: hours
IGF1R: Insulin-like growth factor 1 receptor
MET: MET proto-oncogene, receptor tyrosine-kinase (c-Met or HGFR)
NSCLC: Non-small cell lung cancer
PAR: Parental
qPCR: Quantitative polymerase-chain reaction
SD: Standard deviation
SED: Stepwise-escalating dose
sgRNA: single-guide RNA
TKI: Small molecule tyrosine-kinase inhibitor.
