# Supplementary material for: IGF1R depletion facilitates MET-amplification as mechanism of acquired resistance to erlotinib in HCC827 NSCLC cells
Source: Oncotarget. 2017 Mar 18;8(20):33300–15. doi: 10.18632/oncotarget.16350 (PMC5464869; doi:10.18632/oncotarget.16350)
Supplement: Supplementary file 1 [file oncotarget-08-33300-s001.pdf]

# IGF1R depletion facilitates *MET*-amplification as mechanism of acquired resistance to erlotinib in HCC827 NSCLC cells

## SUPPLEMENTARY DATA

### Genomic knock-out screening by PCR and sanger sequencing

IGF1R genomic knock-out was detected using conventional PCR on cell lysates. Cells were trypsinized with 30 µL in the 96-well plate and stopped with 90 µL of complete medium. Two-thirds of the suspension (80 µL) was passaged for further growth, while the rest (40 µL) was transferred to a PCR tube for lysing. The PCR tubes were centrifuged for 3 min at 1500 rpm, the supernatant was aspirated and pellet re-suspended in 30 µL lysisbuffer (50 mM KCl, 1.5 mM $MgCl_2$, 10 mM Tris/HCL, pH 8.5, 0.5 % Tween 20, 0.5 % Nonidet, 400 µg/mL Proteinase K). The cells were lysed at 65 °C for 30 min followed by 95 °C for 10 min in a thermal cycler. Lysates were stored at −20 °C. Amplification of the target region was performed with HCC827(IGF1R-/-)-PCR-screening primers (Eurofins genomics, Supplementary Table 1). The primers were situated minimum 65 bp from nearest cleavage site giving rise to a *IGF1R*-wildtype amplicon of 300 bp and a HCC827(IGF1R-/-) amplicon of 199 bp. PCR screening was performed in a reaction mixture containing 0.03 units/µL HotStarTaq (Qiagen), 0.2 mM dNTP, 0.4 mM forward primer, 0.4 mM reverse primer, 1X Taq PCR buffer (Qiagen), 2.5 µL cell lysate and $H_2O$ to a volume of 25 µL. The reaction was conducted on a thermal cycler using the protocol: 15 min of heating at 95 °C followed by 40 cycles of denaturation at 95 °C for 15 sec., annealing at assay-specific $T_A$ °C for 15 sec and elongation at 72 °C for 30 sec., and finally one hold at 72 °C for 7 min followed by cooling to 4 °C. The PCR products were evaluated on an agarose gel, and PCR products were purified from the gel using NucleoSpin Gel and PCR clean-up kit (Machery-Nagel) following manufacturer's protocol. Sanger sequencing of the PCR products were performed by GATC Biotech.

### Off-target analysis

*In silico* prediction of potential off-target sites for each sgRNA was performed using http://crispr.mit.edu/. The 4 most likely off-target sites for each sgRNA with a quality score above 0.5 were chosen for investigation. PCR assays were designed for each site with primers (Eurofins Genomics, Supplementary Table 1) covering the potential cleavage site and investigated in both parental cell lines (HCC827 and HCC827(IGF1R-/-)). DNA was obtained from cells by DNA purification using a NaCl precipitation protocol. Briefly, one fourth of a T75 culture flask was scraped and cells were spun for 5 min at 1800 rpm. Cell pellet was washed in PBS and spun again at 1600 rpm for 5 min. The cell pellet was lysed in 500 µL Chorion Villus Lysisbuffer (10 mM Tris, 1 mM EDTA, 150 mM NaCl, 0.5 % SDS, pH 10.5) with 5 µL 20 mg/mL Proteinase K for 2.5 h at 55°C. Samples were mixed with 165 µL 6M NaCl and centrifuged for 15 min at 13000 rpm. Propan-2-ol was added to supernatant in 1:1 volume, and tubes were turned upside down until DNA treads became visible. DNA was captured with glass needles and re-suspended in 100 µL TE buffer. DNA concentration was measured the next day. Conventional PCR amplification was performed using 75 ng purified DNA. A proportion of the PCR products were evaluated on agarose gel and the remaining were purified from the PCR mixture using NucleoSpin Gel and PCR Clean-up kit (Machery-Nagel). Sanger sequencing of the PCR products was performed by GATC Biotech.

### Receptor tyrosine kinase arrays

The Proteome Profiler™ Human Phospho-RTK Array kit (R&D Systems) was used for screening of relative phosphorylation status of 49 key receptor tyrosine kinases following the manufacturer's instructions. The nitrocellulose membranes were blocked for 1 h at room temperature followed by incubation with 300 µg cell lysate at 4 °C overnight with rotation. The next day the membranes were incubated with Anti-Phospho-Tyrosine-HRP Detection Antibody (1:5000) for 2 h at room temperature with rotation. All membranes were developed concurrently using an ImageQuant LAS 4000 system (GE Healthcare Life Sciences) and IQLAS4000 Control Software.

A

| | Exon19 del | T790M | G719X | Ex20Ins |
|---|---|---|---|---|
| **HCC827** | 25.46 | - | 1.12 | 2.46 |
| **HCC827ER** | 24.6 | - | - | 2.04 |
| | | | | |
| **HCC827(IGF1R-/-)** | 25.53 | - | 1.5 | 3.23 |
| **HCC827(IG1F1R-/-)ER** | 24.99 | - | - | 1.15 |

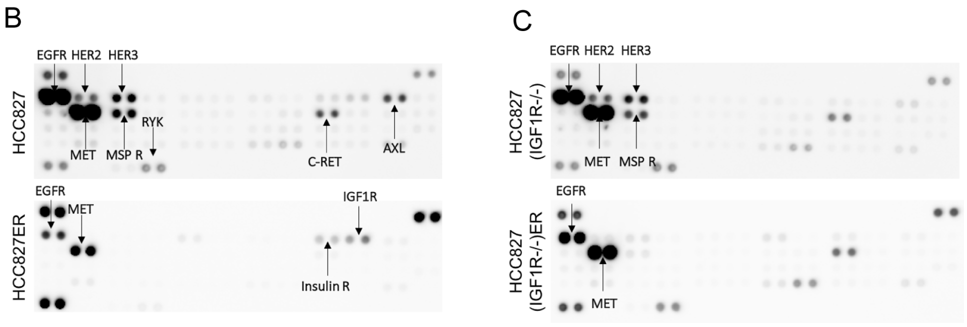


**Supplementary Figure 1: Changes in response to acquired resistance in HCC827. (A).** Output data from Cobas analysis detecting 42 distinct *EGFR* mutations by allele-specific PCR. Output data are provided in arbitrary units: (−) indicate no output and units for G719X and Ex20Ins are below relevancy limit. The results indicate that no novel mutations in *EGFR* occurred during resistance development and that the exon19del was retained in both resistant cell lines. **(B+C)** RTK phosphorylation profile performed on cell lysates from HCC827, HCC827ER, HCC827(IGF1R−/−), and HCC827(IGF1R−/−)ER. Parental cells were pre-treated with 48 hours of DMSO prior to protein harvest. Duplicate reference spots in the corners. Subsets of RTKs are denoted with arrows.

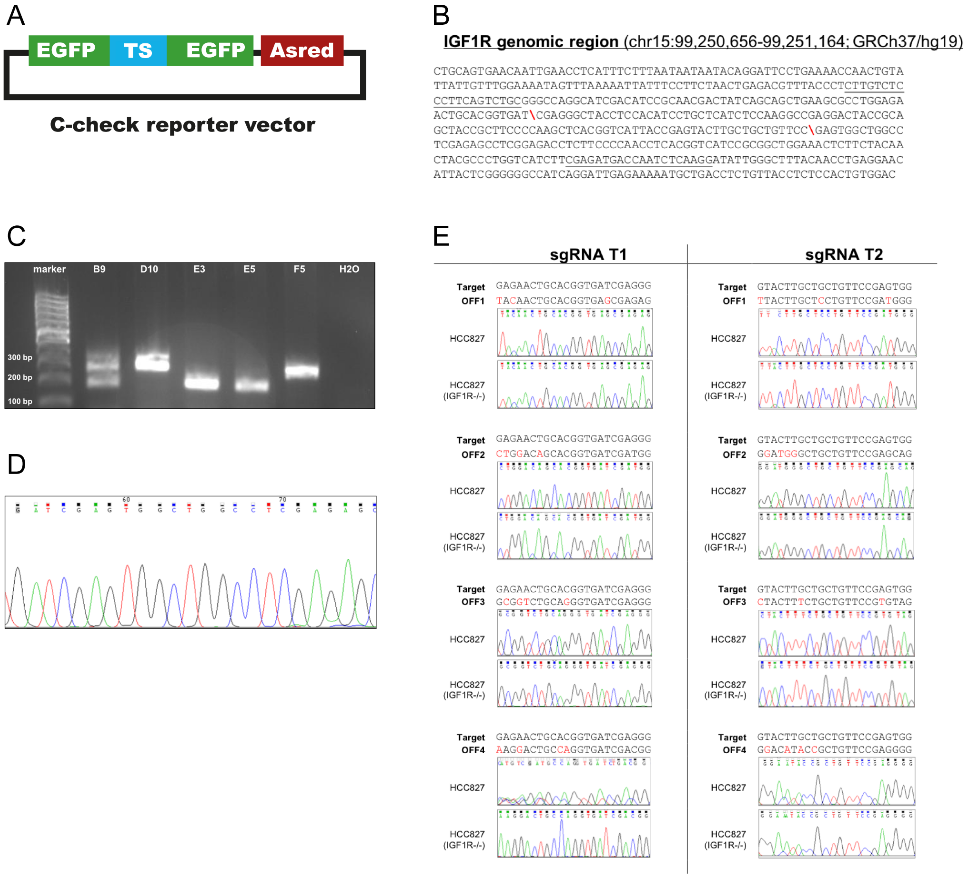


**Supplementary Figure 2: Generation of a HCC827(IGF1R−/−) cell line.** **(A)** Illustration of *IGF1R* C-Check plasmid. The sgRNA target sequence (TS, blue bar) is inserted in the plasmid, here part of *IGF1R* exon 2. When a functional CRISPR/Cas9 system is present in the cells, the sgRNAs will introduce double stranded breaks in TS within the C-Check reporter vector. An intact *EGFP* (green bars) gene will be generated by homologous recombination, and EGFP fluorescence will be detectable. Cells can be sorted according to expression of EGFP to enrich for possible gene-edited cells. AsRED (red bar) is present in the vector to detect transfected cells. **(B)** *IGF1R* genomic region of interest illustrated with sgRNA cleavage sites (red backslashes) and primers for amplification of target region ("HCC827(IGF1R−/−) PCR-screening"). **(C)** PCR screening of gene-edited clones for genetic deletion. *IGF1R* wildtype amplicon is 300 bp and gene edited amplicon is 199 bp if precise end-joining occurs. **(D)** Sanger sequencing of PCR amplicon for validation of genomic knock-out in Clone E3. **(E)** PCR amplification and Sanger sequencing of potential off-target sites for each sgRNA. Target- and potential off-target sequences are aligned with off-sited denoted in red. Sanger sequencing for 4 potential sites per sgRNA investigated in both HCC827 and HCC827(IGF1R−/−).

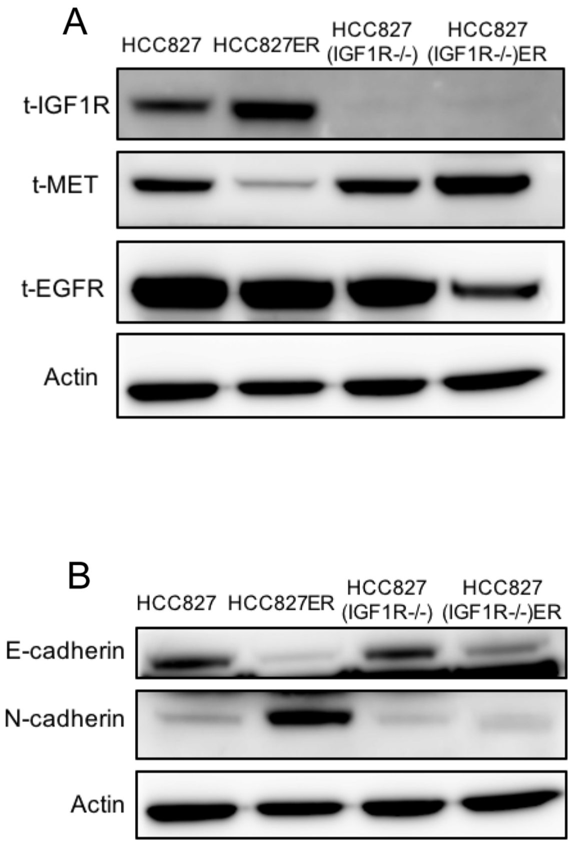


**Supplementary Figure 3: Partial acquisition of EMT features in HCC827ER.** Western blot analysis of **(A)** total amounts of IGF1R, MET, and EGFR protein and **(B)** the cadherin switch from E-cadherin to N-cadherin protein expression in HCC827, HCC827ER, HCC827(IGF1R−/−), and HCC827(IGF1R−/−)ER. β-actin was used as loading control.

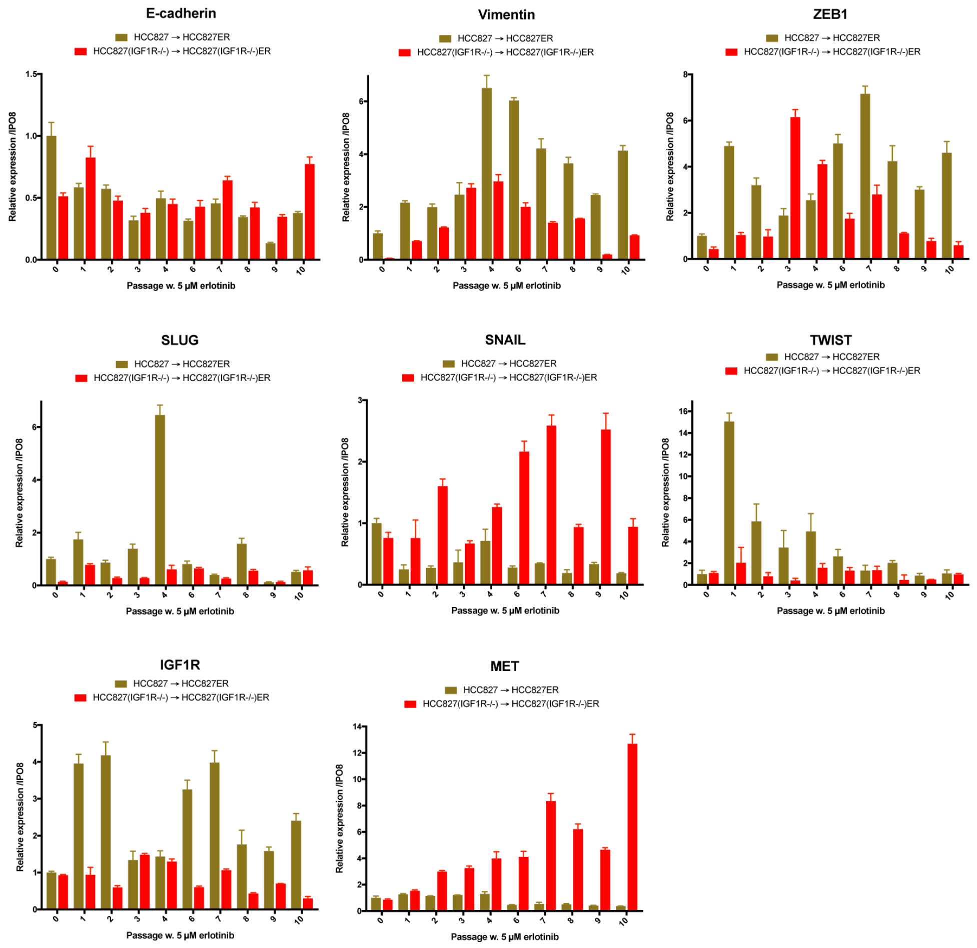


**Supplementary Figure 4: Progressive gene expression changes during resistance development.** mRNA expression level was assessed by qPCR analysis in samples obtained progressively throughout resistance development for HCC827 (beige) and HCC827(IGF1R−/−) (red). Gene expression was normalized to *IPO8* and expression levels for each passage are presented relatively to "HCC827 (beige) passage 0". Expression levels are based on one biological sample and illustrated as mean ± SD.

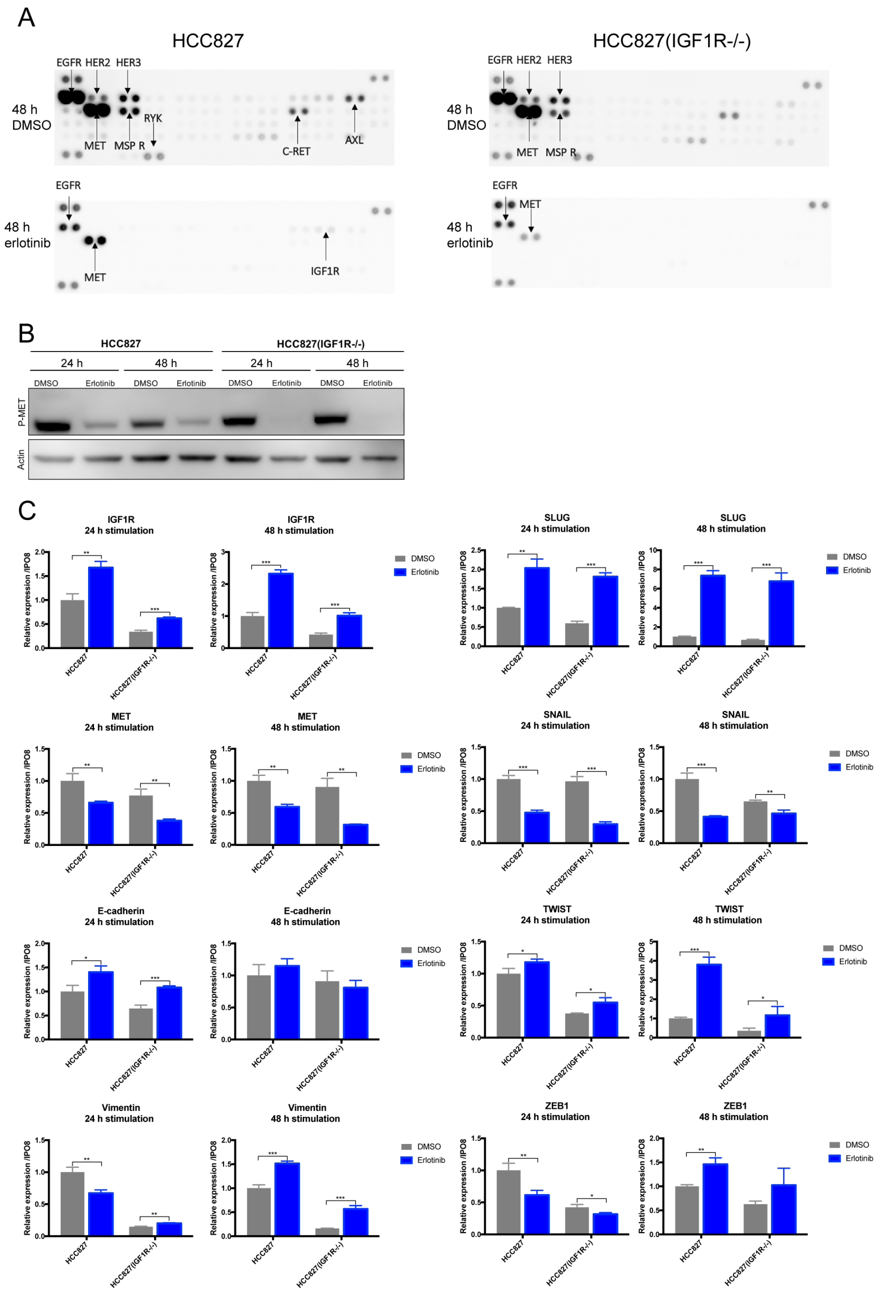


**Supplementary Figure 5: Transient exposure to 5 µM erlotinib.** HCC827 and HCC827(IGF1R−/−) were transiently exposed to 5 µM erlotinib to investigate initial responses to drug exposure. **(A)** RTK phosphorylation profile performed on cell lysates from HCC827 and HCC827(IGF1R−/−) after 48 hours of 5 µM erlotinib or DMSO exposure. Duplicate reference spots in the corners. Relevant RTKs are denoted with arrows. Note the DMSO treated control RTK blots are the same as shown in Supplementary Figure 1. **(B)** Western blot analysis of MET protein phosphorylation (P-MET) after 24 and 48 hours of 5 µM erlotinib (blue) or DMSO (grey) exposure in HCC827 and HCC827(IGF1R−/−). β-actin was used as loading control. **(C)** mRNA expression levels were assessed by qPCR analysis in HCC827 and HCC827(IGF1R−/−) upon 24 and 48 hours of 5 µM erlotinib or DMSO exposure. Gene expression was normalized to *IPO8* and expression levels are presented relatively to HCC827 DMSO treated control for individual targets (at each time point). Gene expression is illustrated as mean ± SD, and significance is calculated for each individual target between DMSO and erlotinib treated cells for each cell line. (*p<0.05, **p<0.01, ***p<0.001).

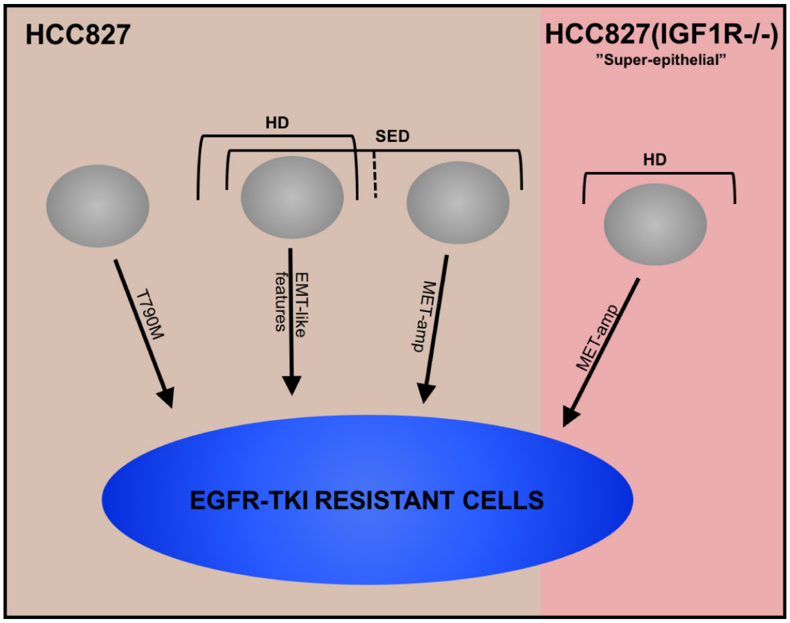


**Supplementary Figure 6: Illustration of resistance mechanism shifts. The cells have the ability to use different mechanisms for obtaining resistance.** Our studies in context with the literature have indicated some mechanisms to occur more often in certain settings than others. Both clinical and *in vitro* established models have developed resistance by acquiring the T790M mutation within EGFR. When HCC827 cells acquire resistance by the stepwise-escalating dose approach (SED) they have repeatedly undergone *MET*-amplification. On the other hand, several cells lines have demonstrated EMT features when made resistant by a high-dose protocol (HD). Our model, HCC827(IGF1R−/−) lacking IGF1R, shifted resistance mechanisms when made resistance through a high-dose approach by a homogenous activation and amplification of the *MET* gene.

**Supplementary Table 1: Primer information for generation of CRISPR/Cas9 system**

| Primer name | Forward (5′-3′) | Reverse (5′-3′) | $T_A$ (°C) | Amplicon size, bp | Sequencing primer |
|---|---|---|---|---|---|
| **HCC827(IGF1R −/−)-PCR-screening** | CTTGTCTCCCTTCAGTCTGC | CCTTGAGATTGGTCATCTCG | 58 | 300 | forward |
| **T1 OFF-T1** | GGAAACATCGTGGGAATCG | AGCCCTTTGGAATGACTGG | 58 | 222 | forward |
| **T1 OFF-T2** | TCTCTGTTTGCAGGCATCG | CCAGAGCCTAGTTCTGAAG | 58 | 201 | reverse |
| **T1 OFF-T3** | AGCAGTGCTGAGGTTGATG | CTATAGCCCTGCCTTTGTG | 58 | 204 | forward |
| **T1 OFF-T4** | AGATGCACGGCAAGAACTG | GGGGTCTTGAAGCACAAC | 58 | 226 | reverse |
| **T2 OFF-T1** | GGAGAGTAGACAGCTTTCC | CCCACAGTCAGTTCAAGTC | 56 | 260 | forward |
| **T2 OFF-T2** | TGAGGTCAGTGGAAAGCTG | CCACTGCCTCTAAATTGGG | 58 | 224 | forward |
| **T2 OFF-T3** | CAATGAGGAGCTGTCGTAG | AACAAACCTGCACATTGTGC | 58 | 251 | forward |
| **T2 OFF-T4** | ATAGAGAAAGGGTGCGGTG | TGAAAGGAGGTGGAGGATG | 58 | 234 | reverse |

**Supplementary Table 2: Primer information for qPCR target genes**

| Target gene | Forward primer (5′-3′) | Reverse primer (5′-3′) | $T_A$ (°C) | Amplicon size, bp |
|---|---|---|---|---|
| ***IGF1R*** | AATAAGCCCCCAAAGGAATG | TGGCAGCACTCATTGTTCTC | 60 | 182 |
| ***MET*** | TGGAGACACTGGATGGGAGT | CAGCGCGTTGACTTATTCAT | 60 | 193 |
| **E-cadherin (*CHD1*)** | GTCCTGGGCAGACTGAATTT | GACCAAGAAATGGATCTGTGG | 58 | 182 |
| **Vimentin (*VIM*)** | GACCAGCTAACCAACGACAAA | TGAAAGATTGCAGGGTGTTT | 58 | 136 |
| ***SLUG*** | GTCCGTCTGCCGCACCTGAG | ACACGGCGGTCCCTACAGCA | 70 | 72 |
| ***SNAIL*** | CGACCACTATGCCGCGCTCT | AGCAGGTGGGCCTGGTCGTA | 68 | 130 |
| ***TWIST*** | GGGCGTGGGGCGCACTTTTA | CGCTGCCCGTCTGGGAATCA | 64 | 70 |
| ***ZEB1*** | AGACATGTGACGCAGTCTGGGT | TGGGCATTCATATGGCTTCTCTCCA | 58 | 129 |
| ***ESRP1*** | GAACTCTTCAGGAGCACAGC | GGCACAAATTGCTGAGGTAG | 58 | 119 |
| ***IPO8*** | GAA GCA GAT GAA GCC ACA CA | CGG GTC TGT CAG GAT TTG AT | 60 | 250 |

**Supplementary Table 3: Antibody information used for western blot**

| Target | Manufacturer | Cat. No. | Species | Blocking buffer/ diluent | Dilution factor | Secondary anti-body | Size (kDa) |
|---|---|---|---|---|---|---|---|
| **t-IGF1R** | Cell Signaling | 3027 | Rabbit | 5 % BSA | 1:1000 | DAKO | 95 |
| **p-IGF1R** | Cell Signaling | 3024 | Rabbit | 5 % BSA | 1:500 | CST | 95 |
| **t-EGFR** | Epitomic | 2235-1 | Rabbit | 5 % skimmed milk | 1:1000 | DAKO | 170 |
| **p-EGFR** | LSBIO | LS-C49399 | Rabbit | 5 % skimmed milk | 1:500 | DAKO | 170 |
| **t-MET** | Cell Signaling | 3127 | Mouse | 5 % skimmed milk | 1:1000 | DAKO | 145 |
| **p-MET** | Cell Signaling | 3129s | Rabbit | 5 % BSA | 1:1000 | DAKO | 145 |
| **E-cadherin** | BD Biosciences | 610182 | Mouse | 5 % BSA | 1:2000 | DAKO | 120 |
| **N-cadherin** | Abcam | ab76011 | Rabbit | 5 % BSA | 1:1000 | DAKO | 100 observed: 140 |
| **Actin** | Sigma | A5316 | Mouse | 5 % skimmed milk | 1:5000 | DAKO | 42 |

| Target | Manufacturer | Cat. No. | Species | Diluent | Dilution factor | Conjugate |
|---|---|---|---|---|---|---|
| **Anti-rabbit** | DAKO | P0448 | Goat | 5 % skimmed milk | 1:4000 | HRP |
| **Anti-mouse** | DAKO | P0447 | Goat | 5 % skimmed milk | 1:4000 | HRP |
| **Anti-rabbit** | CST | 7074 | Goat | 5 % skimmed milk | 1:5000 | HRP |

**Supplementary Table 4: Antibody information used for immunofluorescence staining**

| Target | Manufacturer | Cat. No. | Species | Blocking buffer/ diluent | Dilution factor |
|---|---|---|---|---|---|
| **E-cadherin** | BD Biosciences | 610182 | Mouse | 5 % BSA | 1:1000 |
| **Vimentin** | Abcam | AB20346 | Mouse | 5 % BSA | 1:500 |
| **p-MET** | Cell Signaling | 3129s | Rabbit | 5 % BSA | 1:50 |

| Target | Manufacturer | Cat. No. | Species | Diluent | Dilution factor | Conjugate |
|---|---|---|---|---|---|---|
| **Anti-mouse** | Life Technologies | A11029 | goat | 5 % BSA | 1:2000 | AlexaFlour® 488 |
| **Anti-mouse** | Invitrogen | A21424 | goat | 5 % BSA | 1:2000 | AlexaFlour® 555 |
| **Anti-rabbit** | Life Technologies | A11034 | goat | 5 % BSA | 1:2000 | AlexaFlour® 488 |
